# Strychnia in Paralysis

**Published:** 1845-12

**Authors:** 


### STRYCHNIA IN PARALYSIS.

An interesting case of paralysis occurred under our observation, a few months since, in which strychnia was given with apparent success; we say apparent, because we know how often the relief which ensues upon the use of a remedy is falsely ascribed to the operation of the remedy. The subject of our case was a woman of color, about 55 years of age, whose health for the greater part of her life had been indifferent. For many years she suffered with dysmenorrhœa, attended with obstinate constipation, and the usual constitutional disturbance. About the middle of August while in her ordinary health, except some lameness in her left leg which was thought to arise from a rheumatic affection, she was suddenly seized with paralysis of the left leg and arm, and, as she was standing on the side of a bed at the time adjusting the musquetoe-bar, fell prostrate upon the floor. She was not insensible, however, and was even able to rise and sit on a chair; but, attempting to walk half an hour afterwards, she again fell, and experienced, then, a total loss of power in the leg and arm. She was bled, under the impression that the attack was of an apoplectic character, but we are inclined to think that she derived no benefit from the bleeding, although there was no evidence that it was injurious to her.

Cathartic medicines having been given for a few days she was put upon strychnia, in doses of the sixteenth of a grain, three times daily, and this course was kept up until she had taken six grains. Before she had finished the first vial, containing three grains of the article, she began to recover the use of her arm, the improvement in it being very manifest from day to day. With the strychnia, during the last three weeks of her confinement, she took five grains of the precipitated carbonate of iron three times a day. At the end of six weeks she was able to walk about her room, and has been now for nearly two months about as active as she was before this attack. It is impossible to say that she was relieved by the strychnia, but the case is deemed one of sufficient interest to be placed on record. Certain it is, that it was a highly unpromising one, the age and previous health of the patient being considered. Y.

